# Optimization Ultrasound-Assisted Deep Eutectic Solvent Extraction of Anthocyanins from Raspberry Using Response Surface Methodology Coupled with Genetic Algorithm

**DOI:** 10.3390/foods9101409

**Published:** 2020-10-04

**Authors:** Hongkun Xue, Jiaqi Tan, Qian Li, Jintian Tang, Xu Cai

**Affiliations:** 1 Key Laboratory of Particle & Radiation Imaging, Ministry of Education, Department of Engineering Physics, Tsinghua University, No. 30 Shuangqing Road, Haidian District, Beijing 100084, China; xuehk@mail.tsinghua.edu.cn (H.X.); liqianb116@163.com (Q.L.); jttanglabb116@163.com (J.T.); 2 Academy for Advanced Interdisciplinary Studies, Peking University, No. 5 Yiheyuan Road, Haidian District, Beijing 100871, China; tanjiaq@pku.edu.cn

**Keywords:** raspberry, anthocyanins, ultrasound-assisted deep eutectic solvent extraction, optimization, anthocyanins structure

## Abstract

Raspberries have been reported to contain abundant anthocyanins and other active compounds. To extract anthocyanins from raspberries more efficiently, a novel procedure of ultrasound-assisted deep eutectic solvent extraction (UADESE) was proposed in this paper. The extraction process was optimized by response surface methodology coupled with a genetic algorithm. The optimum extraction parameters to achieve the highest yield of anthocyanins 1.378 ± 0.009 mg/g from raspberry powder via UADESE were obtained at a water content of 29%, ultrasonic power of 210 W, extraction temperature of 51 °C and extraction time of 32 min. The AB-8 macroporous resin combined with the high-speed counter current chromatography (HSCCC) method were further used to isolate and purify the anthocyanins extracts obtained under optimum extraction conditions, and the structure of purified anthocyanins components were identified by UV-Visible spectrophotometer (UV-Vis), high-performance liquid chromatography (HPLC), high-performance liquid chromatography-electrospray ionization-mass spectrometry (HPLC-ESI-MS/MS), ^1^H nuclear magnetic resonance (NMR) and ^13^C-NMR spectra. The two anthocyanins (cyanidin-3-glucoside with a purity of 92.25% and cyanidin-3-rutinoside with a purity of 93.07%) identified were consistent with those present in raspberries. These findings provided an effective and feasible method for extraction, isolation and purification of anthocyanins from natural plant resources.

## 1. Introduction

Raspberries (*Rubus idaeus* L.) are grown all over the world, including North America, Japan, Russia and Northeast China. Raspberry cultivation is deeply favored because of its high nutritional value, unique aroma and attractive red pigment [1]. The raspberry is a good source of bioactive compounds, including anthocyanins, flavanols, various vitamins, superoxide dismutase and phenolic acids [2]. Anthocyanins, as one of the most essential active components in raspberries, are water-soluble natural pigments with a strong resistance to oxidation, responsible for reduction of the risk of cardiovascular disease, cancer, chronic inflammation and ageing, as well as the improvement of immunity and protection of eyesight [3]. Anthocyanins extracted from raspberries have good stability, nontoxic side effects and strong coloring power, which can be used in the food and medicinal industries. Finding the most efficient, green and environmentally friendly extraction methods for the isolation of anthocyanins from raw plant materials has been a challenging task in the last few decades.

Previously, anthocyanin extraction from natural plant resources used conventional solvent (methanol, ethanol, ethyl acetate, etc.) extractions, which caused excessive solvent consumption and environmental pollution [4]. Recently, the use of deep eutectic solvents (DESs) as the green alternative to conventional solvents has been greatly expanded. DESs, as the new generation of liquid salts, are usually based on mixtures of relatively cheap and easily available components. For example, nontoxic hydrogen bond acceptors (HBA, e.g., choline chloride) with naturally derived uncharged hydrogen bond donors (HBDs, e.g., glucose, 1,3-butanediol, 1,4-butanediol, glycol and glycerol) provide “green profiles” and have broad application prospects in the field of green technologies [5]. Since their emergence, DESs as potential green solvents have attracted extensive attention in various industrial fields, including the extraction of bioactive compounds from different natural plant sources. Previous researches have reported the applications of DESs in the extraction of phenolic compounds [6,7,8]. Furthermore, another principle of green extraction is reduced energy consumption by using potential innovative technologies—for instance, ultrasound. Ultrasound-assistant extraction (UAE) may provide a vital energy source to enhance extraction. The UAE has many merits, e.g., higher yield; shorter extraction time and lower solvent consumption, temperature and production costs in comparison with the conventional solvent extractions [9]. In addition, the low temperature and the short extraction time of UAE can avoid the degradation of anthocyanins caused by high temperature, which contributes to the improvement of the anthocyanin yield [10]. Thus, the UAE method is used in the extraction of bioactive compounds from different natural plant sources [11,12,13]. The combination of UAE and DESs extraction methods has the advantages of both methods, thereby providing effective and environmentally friendly alternatives to the traditional anthocyanin extraction methods. Nevertheless, the ultrasonic-assisted deep eutectic solvents extraction (UADESE) is yet to be reported for raspberries. Hence, the UADESE method is used to extract anthocyanins from raspberries to achieve the maximum yield of anthocyanins in this study.

The yield of bioactive compounds from different natural plant sources depends on many factors that affect the extraction process. These factors include solvent properties, extraction temperature, extraction time, nature of the plant material and other parameters. Thus, optimizing the extraction conditions is essential to achieve the highest yield of anthocyanins from raspberries. The response surface methodology (RSM) is a mathematical and statistical method that is widely used to investigate the individual and the interaction effects of these factors on the yield of the target compounds [14]. Additionally, RSM can effectively diminish the total number of experimental trials and reduce the experiment cost and time. As such, RSM is successfully used in enhancing and optimizing extraction processes. However, RSM has a high requirement for the selection of experimental points. If the selection of experimental points is not appropriate, RSM will get less than ideal optimization results. The genetic algorithm (GA) has the characteristics of global optimization, which can achieve improved prediction and optimization effects [15]. The combination of RSM and GA can avoid RSM defects and give full play to the advantages of GA in global optimization. Therefore, this method is used to optimize the extraction conditions for the UADESE anthocyanins from raspberries.

Presently, the separation and purification of anthocyanins mainly use traditional methods, such as solid-liquid extraction, column chromatography and adsorption, etc. [16,17]. These methods have not been widely used due to low efficiency, high solvent consumption and long reaction periods. High-speed counter current chromatography (HSCCC), as a liquid-liquid partition chromatography technique, has excellent separation efficiency and high sample recovery, which is widely employed for the separation and purification of polyphenolic compounds, anthraquinones and alkaloids [18,19,20]. However, the separation and purification of anthocyanins from raspberries by UADESE has not yet been reported. The AB-8 macroporous resin combined with the HSCCC method is used to separate and purify anthocyanins from raspberries in this study.

This study aims to: (1) optimize the extraction conditions for the UADESE of anthocyanins from raspberries using RSM coupled with GA and (2) identify the structure of the purified anthocyanins by UV-Vis, HPLC, HPLC-ESI-MS/MS, ^1^H NMR and ^13^C-NMR spectra.

## 2. Materials and Methods

### 2.1. Experimental Materials

Raspberries were collected from Heilongjiang Raspberry Technology Development Co., Ltd. (Heilongjiang Province, China) in August 2019. Raspberries were pureed using a Y91S household electrical grinder (Jiuyang Co., Ltd., Hangzhou, Zhejiang Province, China), and dried in an FD-1A-50 freezing-vacuum dryer (Jiangsu Yutong drying Engineering Co., Ltd., Jiangsu Province, China) at −18 °C until the moisture content was <5%, and then, the dehydrated raspberry jam was stored at −18 °C for 12 h before crushed. The frozen samples were thawed and milled into powdered particles smaller than 0.45 mm using an electric mill (HK-600, Hongke Food Machinery Co., Ltd., Yongkang, Zhejiang Province, China), sealed in a brown reagent bottle and stored at 4 °C for the follow-up experiments.

### 2.2. Reagents and Solvents

Cyanidin-3-O-glucoside (C3G) was purchased from Chengdu Gelip Biotechnology Co., Ltd. (Chendu, China). Choline chloride, glycerol, 1,3-butanediol, 1,4-butanediol, glycol, EtOH and sodium acetate buffer of analytical purity were afforded from Shanghai McLean Biochemical Technology Co., Ltd. (Shanghai, China). AB-8 macroporous resin was offered from Tianjin Bohong Resin Technology Co., Ltd. (Tianjin, China). Formic acid, potassium chloride buffer and hydrochloric acid were from Shanghai Jinjinle Industrial Co., Ltd. (Shanghai, China). BuOH, acetonitrile (ACN), trifluoroacetic acid (TFA) and methyl tert butyl ether (MTBE) were obtained from Nanjing Huijing Petrochemical Co., Ltd. (Nanjing, China).

### 2.3. Preparation of DESs

In this study, 10 types of DESs were prepared following a previous research, with some modifications [21]. Typically, the HBA (choline chloride) and HBDs (glucose, 1,3-butanediol, 1,4-butanediol, glycol and glycerol) were well-mixed in a proper molar ratio and then subjected to continuous stirring using a magnetic stirring apparatus (MS-H280-Pro, Jinan Laibao Medical Equipment Co., Ltd., Jinan, Shandong Province, China) at 80 °C, resulting in the formation of a homogeneous and transparent liquid, which is denoted as the DESs. The chemicals used, the molar ratio and the ratio of DESs to water are shown in Table 1.

### 2.4. UADESE Procedures

Anthocyanins were extracted using UADESE in accordance with a previously described method, with some modifications [22]. The lyophilized raspberry powders were mixed with the prepared DESs under different solid-to-liquid ratios and carried out using RY-TQ-C ultrasound equipment (Beijing, China). The ultrasonic power, extraction time and temperature were set through the control panel of the ultrasonic equipment. Thereafter, the extracted samples were centrifuged at 3000× *g* and 25 °C for 15 min by using a KH20R-II centrifuge from Hunan Kaida Scientific Instrument Co., Ltd. (Changsha, Hunan Province, China), and then, the supernatants obtained were filtered by using a 0.22-μm nylon filter membrane (Tianjin Jinteng Experimental Equipment Co., Ltd., Tianjin, China). The filtrates were concentrated via a DZFY-2L rotary evaporator from Shanghai Yaote Instrument Equipment Co., Ltd. (Shanghai, China) at 40 °C and freeze-dried by an FD-1A-50 dryer (Beijing Biocool Experimental Equipment Co., Ltd., Beijing, China) for 48 h. After drying, the crude anthocyanin powders were sealed in a brown reagent bottle and stored at −18 °C.

### 2.5. Determination of the Anthocyanin Yield

The yield of anthocyanins from raspberries was measured by a pH differential method [23]. In short, 3 mL of anthocyanins extracts were diluted with 5 mL of two different buffers (0.025-M KCl-HCl buffer at pH 1.0 and 0.4-M CH_3_COONa-HCl buffer at pH 4.5). These mixtures were stored away from light for 30 min at 25 °C. The absorbance of each sample was determined at 510 and 700 nm using a UV-3802S visible spectrophotometer (Hangzhou, Zhejiang Province, China), respectively. The anthocyanin yield was calculated by Equation (1).

(1) $$c=\frac{\left[\left(A_{510}-A_{700}\right)pH_{1.0}-\left(A_{510}-A_{700}\right)pH_{4.5}\right]\times M_{\omega }\times DF\times V\times 1000}{\varepsilon \times L\times m}$$

where *M_ω_* is the molecular weight of C3G (449.2 g/mol), *DF* is the dilution factor, *ε* is the molar extinction coefficient of C3G (26,900 L/mol·cm^−1^), *L* is the path length (cm), *V* is the total volume of the extraction solvent (mL) and *m* is the weight of the raspberry powder (g).

### 2.6. Experimental Design

#### 2.6.1. Screening of Variables

Choosing a suitable range of experimental factors contributes to the improvement of the yield of anthocyanins from raspberries. In this study, the factors and their ranges were selected on the basis of our pre-experiment results. Too-high or too-low experimental factor levels were not conducive to the extraction of anthocyanins, according to the pre-experiment results. Thus, the selection of the experimental factor ranges are as follows: The water content in DESs of 10–50%, ultrasonic power of 100–500 W, extraction temperature of 40–60 °C, extraction time of 10–50 min and solid-to-liquid ratio of 1:10–1:30 g/mL. These experimental factors were initially performed to single-factor experiments. Each single-factor experiment was repeated thrice, and the experimental results were presented as mean ± standard deviation (SD).

#### 2.6.2. Box-Behnken Design (BBD)

According to the single-factor experimental results, the Box-Behnken design (BBD) of 4-factor-3-level was conducted to investigate the influence of different variables and their interactions on the yield of anthocyanins from raspberries. The following four independent factors were considered: water content in DESs (*X*_1_), ultrasonic power (*X*_2_), extraction temperature (*X*_3_) and extraction time (*X*_4_) were selected as independent variables, whereas the anthocyanin yield (*Y*) is regarded as the response. The level and code of these factors are shown in Table 2. All experiments were performed in triplicate. The experimental data was fitted into the full second-order polynomial model to obtain the regression coefficients, as presented in Equation (2) [24].

(2) $$Y=\beta_{0}+\overset{k}{\underset{j=1}{\sum }}\beta_{j}X_{j}+\overset{k}{\underset{j=1}{\sum }}\beta_{jj}X_{j}^{2}+\underset{i}{\sum }\overset{k}{\underset{<j=2}{\sum }}\beta_{ii}X_{i}X_{j}+e_{i}$$

#### 2.6.3. Genetic Algorithm (GA)

The GA is a stochastic nonlinear optimization algorithm based on the mechanics of natural genetics and natural selection. The basic process of GA is shown in Figure S1. The RSM model was employed as the fitness function for the GA to optimize the extraction parameters of anthocyanins from raspberries. The GA was carried out as follows. The Gaussian mutation function was chosen to add a random number to each individual vector entry. The rank scale function was used to fit the individuals on the basis of the original scores. Other functions selected in the optimization process included the random uniform selection function and decentralized cross-function. The GA toolbox of the MATLAB version R2018b (The MathWorks, Inc., Natick, MA, USA) was employed in optimization studies. The objective function can be detected as the following Equation (3):

(3) $$\mathrm{Maximize}\;y=f\left(x\right);\;X_{I}^{L}\leq X_{i}\leq X_{i}^{U},\;i=1,2,\ldots ,n$$

where *f*(*x*) is the objective function obtained from the RSM model, *x* is the input vector, *y* is the anthocyanins yield (mg/g), *n* is the number of experimental factors and *X_i_^L^* and *X_i_^U^* are the lower and upper bounds of *X_i_*, respectively.

### 2.7. Purification of Anthocyanins Using Macroporous Adsorption Resin

The separation and purification of anthocyanins were described as previously reported [25]. The anthocyanin extracts obtained under the optimal extraction process (200 mL) were slowly added to a pretreatment AB-8 resin-column (2.6 cm × 60 cm) at a flow rate of 3 BV/h (BV, Bed volume). The column that absorbed the anthocyanins was eluted with ultrapure water at 4 BV/h to remove the impurities, including sugars, proteins and polar compounds, followed by elution with 70% ethanol (*v*/*v*) of pH 3 at 2 BV/h. The eluent was collected, and the ethanol was recovered by a rotary evaporator at 40 °C to obtain an anthocyanin-rich solution. The anthocyanin-rich solution was further freeze-dried using an FD-1A-50 dryer to obtain the purified anthocyanin extracts powder.

### 2.8. High-Speed Counter Current Chromatography Isolation of High-Purity Anthocyanins

#### 2.8.1. Measurement of the Partition Coefficient (K)

In the present study, different volume ratios of BuOH, MTBE, ACN, water and TFA were prepared and equilibrated in a separatory funnel, and then, the purified anthocyanins extract powders (10 mg) were dissolved in the solvent at 25 °C. Five milliliters of each phase was evaporated to dryness. The above residues were dissolved by 5-mL methanol and then analyzed by a UV-Visible spectrophotometer at 254 nm. Finally, the *K* value is the peak area of the fraction in the upper phase (A_upper_) divided by that in the lower phase (A_lower_): *K* = A_upper_/A_lower_.

#### 2.8.2. Preparation of High-Speed Counter Current Chromatography Solvent System and Sample Solution

The solvent system composed of BuOH-MTBE-ACN-water-TFA (2:2:1:5:0.01, *v*/*v*/*v*/*v*/*v*) was prepared by adding the solvents to a separation funnel in a certain ratio, vigorously mixed and allowed to equilibrate at 25 °C. The two phases were separated shortly before use. The lower phase was used as the mobile phase, while the upper phase was employed as the stationary phase. Three hundred milligrams of the purified anthocyanin extract powders were dissolved in a 20-mL solvent mixture, which was composed of 10-mL lower phase and 10-mL upper phase. The samples were further separated by a TBE-300C HSCCC system (Shanghai Tauto Biotechnique Co., Ltd., Shanghai, China).

#### 2.8.3. High-Speed Counter Current Chromatography Separation Procedure

The multilayer column was first filled entirely with the upper phase, and the mobile phase was then pumped into the column at a flow rate of 2.0 mL/min, while the apparatus was rotated at 900 rpm. After a clear mobile phase eluted at the tail outlet and the hydronamic equilibrium was reached, crude samples were then injected into the injection valve. The column effluent was continuously monitored by UV detection at 254 nm. Fractions were collected into test tubes at 5-min intervals per tube by automatic fraction collectors and then evaporated. Each fraction was collected according to the chromatogram. After the separation, the retention of the stationary phase (*S_r_*) was expressed as a percentage of a volume of the stationary phase (*V_s_*) relative to the total column capacity (*V_c_*), which was *S_r_* = (*V_s_*/*V_c_*) × 100%.

### 2.9. High-Speed Counter Current Chromatography Peaks Identification

The peaks from the HSCCC of the purified anthocyanin extract powders were identified by UV-Vis, HPLC, HPLC-ESI-MS/MS, ^1^H NMR and ^13^C NMR spectra.

#### 2.9.1. UV-Visible Spectrophotometer (UV-Vis) Analysis

The fractions obtained by using HSCCC were dissolved in 0.03% (*v*/*v*) potassium chloride solution and were scanned in the wavelength range of 200–700 nm to analyze the spectral characteristics.

#### 2.9.2. High-Performance Liquid Chromatography-Diode Array Detector (HPLC-DAD) and High-Performance Liquid Chromatography-Electrospray Ionization-Mass Spectrometry (HPLC-ESI-MS/MS) Analysis

The HPLC-DAD and HPLC-ESI-MS/MS methods for the analysis of the components obtained by HSCCC were basically consistent with previous studies. Before the analysis, the components were dissolved in 5-mL HCl–methanol solution (0.1% (*v/v*)) and filtered through a 0.45-μm filter membrane. The 1100 series liquid chromatography system equipped with a DAD and Zorbax Eclipse XDB-C_18_ column (4.6 mm × 150 mm, 5 μm, Agilent, Santa Clara, CA, USA) was employed to determine the components contents. Two solvents, including 5% (*v*/*v*) formic acid (mobile phase A) and 1% (*v*/*v*) formic acid-acetonitrile (mobile phase B) were used in the mobile phase. The gradient elution was as follows: 5–20% B (0–5 min), 20–25% B (5–20 min), 25–30% B (20–30 min), 30–33% B (30–35 min) and 33–5% B (35–40 min). The mobile phase was pumped by the system at a rate of 0.8 mL/min. The injection volume of the sample was set to 20 µL. Moreover, the column temperature and wavelength were 25 °C and 520 nm, respectively. The purity of the components can be calculated by Equation (4) [26].

(4) $$w_{i}\left(\%\right)=\frac{f_{i}A_{i}}{\sum^{\;}f_{i}A_{i}}$$

where *w_i_* and *A_i_* are the purity and peak area of component *i*, respectively. *f_i_* is the correction factor.

The mass spectra were obtained at a range of 100–1000 *m/z* in the positive mode [27]. The voltages of the capillary, sampling cone and extraction cone were set at 2.0 kV, 40 V and 2.0 V, respectively. The temperatures of the source and desolvation were set to 115 °C and 350 °C, respectively. The time of the scan and interscan were 13 min and 0.28 s, respectively. The gas flow of the cone and desolvation were 50 and 900 L/h, respectively. The collision energy was 20.0–45.0 eV. The Mass-Lynx TM V 4.1 software (Waters Corp., Milford, MA, UK) was used to collect the experimental data.

#### 2.9.3. Nuclear Magnetic Resonance (NMR) Analysis

^1^H and ^13^C-NMR spectra were recorded on a Bruker Advance III 400 spectrometer (Bruker, Karlsruhe, Germany). The components were dissolved in CD_3_OD. The structure of the anthocyanins was determined by comparing the experimental results with those reported in the literature.

### 2.10. Statistical Analysis

All experimental results were represented as the mean ± SD. The one-way analysis of variance was used to analyze the statistical differences between groups. *p <* 0.05 represented the experimental data with statistical significance.

## 3. Results and Discussions

### 3.1. Screening of DESs System for the Extraction of Anthocyanins

**Figure 1:**
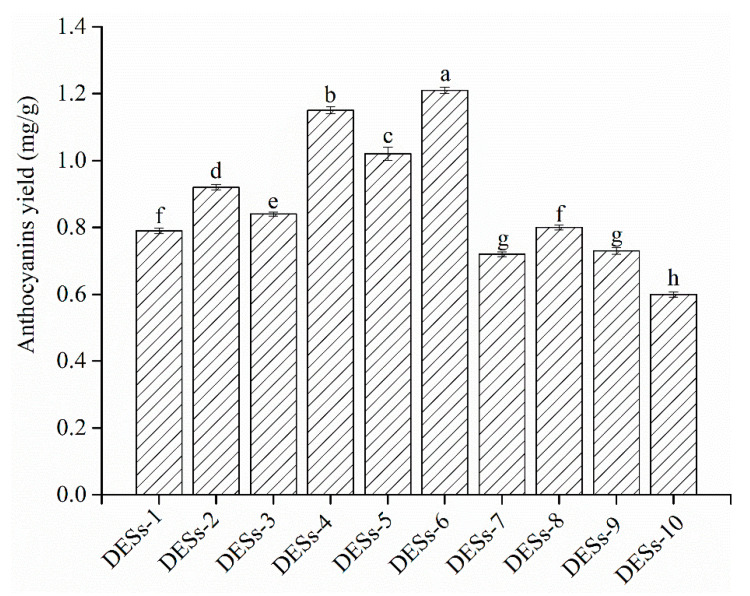
Effect of different deep eutectic solvents (DESs) on the yield of anthocyanins from raspberries. The different lower-case letters indicate significant differences (*p* < 0.05).

Different types of DESs (HBA: choline chloride and HBD: glycerol, 1,3-butanediol, 1,4-butanediol, glycol and glucose) have different physicochemical properties, such as solubility, viscosity, density and conductivity, which have an important effect on the anthocyanin yield and the extraction efficiency [28]. Consequently, choline chloride as the HBA and five types of HBDs (glucose, 1,3-butanediol, 1,4-butanediol, glycol and glycerol) were chosen to prepare DESs in this study. Figure 1 describes the yield of anthocyanins from raspberries extracted with 10 types of DESs. The results indicated that the best extraction anthocyanin yield followed the order: DESs-6 > DESs-4 > DESs-5 > DESs-2 > DESs-3 > other DESs (DESs-1, 7−10), which might be because the extraction efficiency depended on the polarity of the anthocyanins. The polarity of DESs-6 was similar to that of the anthocyanins. According to the rule “like dissolves like”, anthocyanins were highly prone to dissolving in DESs-6 [29]. Moreover, DESs-6 had a lower viscosity and surface tension, improving the permeability to cells for dissolving anthocyanins [29]. The effects of DESs-6 and DESs-4 on the anthocyanins yield showed that the position of the hydroxyl in the polyol also affected the viscosity and the polarity of the DESs, thereby affecting the extraction efficiency. At the same HBD, the DESs with a mole ratio of 1:3 had a lower viscosity and surface tension than DESs with a mole ratio of 1:2, which could easily extract the solvent penetration and promote the dissolution of anthocyanins from raspberries. Therefore, DESs-6 (1,4-butanediol as the HBD and mole ratio of 1:3) was selected as the best extraction solvent to carry out the subsequent experiments.

### 3.2. Single-Factor Experiments for Anthocyanin Extractions

**Figure 2:**
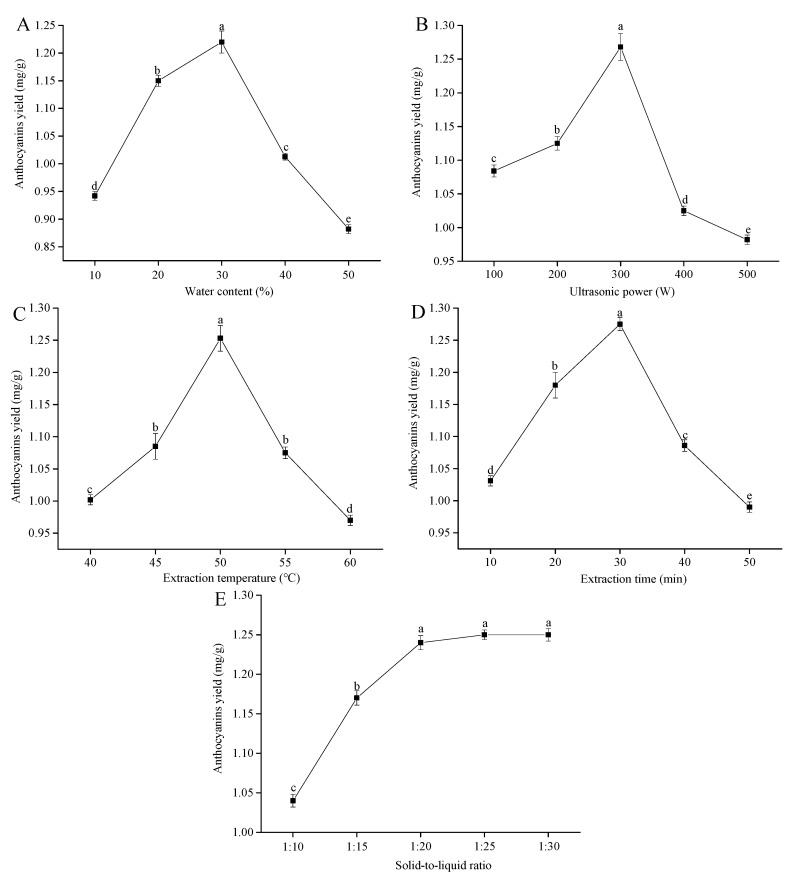
Effects of different extraction variables on the anthocyanin yields from raspberries. (**A**) Water content, (**B**) ultrasonic power, (**C**) extraction temperature, (**D**) extraction time and (**E**) solid-to-liquid ratio. Note: The different lower-case letters indicate significant differences (*p <* 0.05; the same below).

The water content can change the physicochemical properties of DESs, especially the viscosity and polarity [30]. Choosing the suitable water content in DESs is beneficial to improve the mass transfer rates and the anthocyanin yields. Thus, the effects of different water contents (10%, 20%, 30%, 40% and 50%) on the yield of anthocyanins from raspberries was further investigated. The results are shown in Figure 2A. The yield of anthocyanins memorably increased at water contents of 10–30% (*p <* 0.05). This effect might be because the addition of water effectively weakened the hydrogen bonding of the DESs [31], resulting in a rapid decrease in viscosity, which was conducive to improving the solubility and the mass transfer rate of the anthocyanins. Nevertheless, the anthocyanin yields observably decreased when the water content continuously increased from 30% to 50% (*p <* 0.05), which was because the excessively high water content increased the DESs polarity and decreased the solubility of anthocyanins in the DESs [32]. Thus, 20%, 30% and 40% water contents were employed for the subsequent experiments.

Increasing studies have confirmed that the ultrasound power could significantly affect the yields of anthocyanins [33]. Hence, experiments were performed to investigate the effects of the ultrasound power on the anthocyanin yields. The yields of anthocyanins were obviously enhanced with increasing of the ultrasound power from 100 to 300 W (*p <* 0.05) and reached the maximum anthocyanin yields of 1.268 ± 0.020 mg/g at 300 W (Figure 2B). The reason for this phenomenon was that ultrasonic waves generated a cavitation effect and localized pressure in the solvent, causing a faster movement of the molecules and improved penetration of the solvent into the raspberries, which promoted the release of intracellular anthocyanins into the solvent and improved the anthocyanin yields [34]. However, once the ultrasound power was over 300 W, the anthocyanin yields substantially declined (*p <* 0.05), which was attributed to the reason that high-ultrasound power produced excessive heat generation in the reaction system. This heat generation resulted in the degradation of the anthocyanins [35]. Consequently, 100, 200 and 300 W of ultrasound power were used for the subsequent experiments.

The extraction temperature is one of the vital parameters that influences the yield of anthocyanins from raspberries. Figure 2C presents the effects of the extraction temperature on the anthocyanin yields. The yields of anthocyanins substantially improved with the increasing extraction temperature, up to maximum values of 1.253 ± 0.020 mg/g (*p <* 0.05). At 50 °C, a marked decrease in the anthocyanins yields was observed (*p <* 0.05). Initially, the reason might be due to the elevated temperature, which could improve the solubility of anthocyanins in the extraction solvent and accelerate the mass transfer of intracellular anthocyanins [36]. However, a high temperature might cause the degradation of anthocyanins because of their thermal susceptibility [37]. Similar experimental results were obtained by other authors in the case of anthocyanins from *Hibiscus* *sabdariffa calyces* for natural food colorants and *Aronia melanocarpa* skin waste by using different temperatures [38,39]. Therefore, the extraction temperatures of 45 °C, 50 °C and 55 °C were used in subsequent experiments.

Many studies have shown that the extraction time could affect the yields of anthocyanins. Experiments were conducted at various extraction times to investigate its influence on the extraction process. Figure 2D shows the yields of anthocyanins initially remarkably enhanced with increasing extraction times up to 30 min (*p <* 0.05), which was attributed to the reasons that, in the early stage of extraction, the intracellular anthocyanins had low diffusion resistance due to ultrasound-damaged cell structures, which would be conducive to anthocyanin extraction [40]. Nevertheless, a prominent decrease in the yield of anthocyanins from raspberries was observed when the extraction time was over 30 min (*p <* 0.05). This could be hinting that excessive extraction times led to the oxidative degradation of anthocyanins [41]. Hence, the extraction times used for the subsequent experiments were 20, 30, and 40 min.

A large number of studies have shown that the solid-to-liquid ratio could impact the yields of anthocyanins. Figure 2E describes the anthocyanin yields dramatically increased when the solid-to-liquid ratio was in the range of 1:10–1:20 g/mL (*p <* 0.05). The reason for this phenomenon was that the solid-liquid contact area and concentration gradient increased with the increase of the amount of extraction solvent, which was conducive to the diffusion of anthocyanins from inside the extraction solvent, resulting in improving anthocyanin yields [42]. Similar results were reported in the case of anthocyanins from blueberry wine pomace by using ultrasonic-assisted extraction [43]. Nevertheless, there was no significant change in anthocyanin yields when the solid-to-liquid ratio increased from 1:20 to 1:30 g/mL (*p >* 0.05). In addition, an excessive extraction solvent might increase the difficulty and cost of post-treatment. Thus, the solid-to-liquid ratio was determined as 1:20 g/mL, and the subsequent optimization was not carried out.

### 3.3. Modeling of the Extraction Process

The experimental design based on RSM coupled with BBD and the data of 29 runs is shown in Table 3, and the variance analyses of the experimental results are shown in Table 4. The experimental results showed that the linear coefficient of the water content (*X*_1_) and extraction temperature (*X*_3_) were extremely significant (*p <* 0.01), whereas the extraction time (*X*_4_) was considerable (*p <* 0.05). In addition, the water content (*X*_1_), ultrasonic power (*X*_2_), extraction temperature (*X*_3_) and extraction time (*X*_4_) involved in the experiment had an extremely remarkable quadratic effect for the anthocyanin yields (*p <* 0.01). The interaction terms of *X*_1_*X*_4_ and *X*_2_*X*_3_ had a highly prominent effect on the anthocyanin yields (*p <* 0.05). The other interaction parameters had no notable effects on the anthocyanin yields (*p >* 0.05). Nonsignificant factors were initially removed, and a multiple regression analysis was used on the experimental data. The predicted anthocyanin yield was calculated by using Equation (5).

(5) $$Y=1.32-0.028X_{1}+0.036X_{3}+0.02X_{4}+0.056X_{1}X_{4}+0.037X_{2}X_{3}-0.12X_{1}^{2}-0.038X_{2}^{2}-0.10X_{2}^{2}-0.045X_{2}^{2}$$

The *p*-value and the *F*-value were employed to evaluate the importance of each parameter. A low *p*-value and high *F*-value suggested that the related experimental factors were highly remarkable. Jambo et al. (2019) confirmed that the model was conspicuous and could promote the experimental factors when the *p*-value was less than 0.01 [44]. The relationship between the description of the above regression equation and the response face value was extremely marked (*p <* 0.0001). However, the lack of fit was not prominent (*p =* 0.7302 > 0.005). The total (*R*^2^) and the adjusted (*R*^2^_adj_) determination coefficients in the regression equation were 0.9369 and 0.8737, respectively, which implied a high relevance between the experimental and the predicted values (Table 4). Consequently, the regression equation adequately represented the real relationship between the experimental factors and the anthocyanin yields and could be employed to obtain the optimal extraction parameters for the UADESE of anthocyanins from raspberries.

Figure S2A shows that the predicted values were consistent with the actual values, indicating that the predicted values could reasonably explain the experimental values. As shown in Figure S2B, these values also followed the normal distribution and had no deviation from the variance. In addition, the plots of the residuals and all data points were within ± 3 (Figure S2C).

### 3.4. Interaction of Process Variables

**Figure 3:**
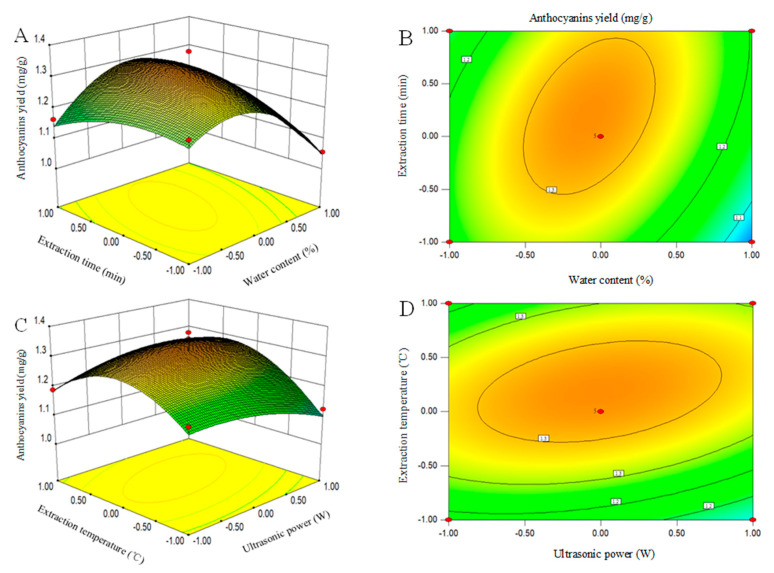
Three-dimensional response surface plots and corresponding contour plots. Influence of the water content and ultrasonic power (**A**,**B**) and ultrasonic power and extraction temperature (**C**,**D**) on anthocyanin yields from raspberries.

The RSM chart shows the interactions between different experimental factors in the extraction process and tests the influence of the interaction between the other two factors on the anthocyanin yields when the other factors are set at the zero level [45]. A 3D response surface figure and 2D contour plots are drawn in accordance with regression Equation (5). The interactions of the experimental factors are described in Figure 3. The 3D response surface could directly reflect the effects of the interactions of various experimental factors on the yields of anthocyanins in raspberries. The steep 3D response surface showed that this experimental factor had a great influence on the yields of anthocyanins, and the interactions of the two experimental factors were highly marked. In the 2D contour, the oval and the circular contours revealed that the interactions between the two factors were significant and not significant, respectively. Figure 3A shows the anthocyanin yields as a function of the water content (*X*_1_) and extraction time (*X*_4_) when the ultrasonic power (*X*_2_) and extraction temperature (*X*_3_) were fixed at a zero level. A marked interaction (*p <* 0.01) was observed between the water content (*X*_1_) and extraction time (*X*_4_) (Table 4). The anthocyanin yields initially increased with the increase of the water content (*X*_1_) and extraction time (*X*_4_) and then decreased. The interactions caused by both the water content (*X*_1_) and extraction time (*X*_4_) in this study were in good agreement with the previous study [46]. Figure 3C clearly depicts that the anthocyanin yields were influenced by both the ultrasonic power (*X*_2_) and extraction temperature (*X*_3_). Moreover, Table 4 indicates a noteworthy interaction between these variables at *p <* 0.05. The yields of anthocyanins also increased and reached the maximum when the ultrasonic power (*X*_2_) and extraction temperature (*X*_3_) increased. Any further increase in these two variables showed a negative effect on the anthocyanin yields. Bosiljkov et al. (2017) observed a similar trend when they investigated the effects of the UADESE conditions on the yields of anthocyanins from wine lees [47].

### 3.5. Optimal Values from the Genetic Algorithm and Confirmation

**Figure 4:**
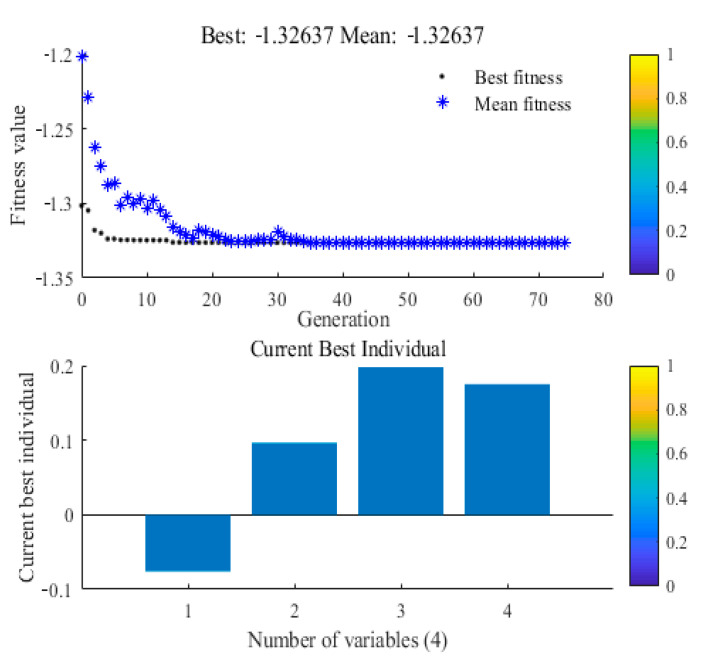
Best fitness and best individual graph.

The GA is employed to achieve a global solution for the multiple regression equation based on RSM. The GA toolbox of the MATLAB version R2018b was used in optimization studies. The running results of the GA M file are shown in Figure 4. The optimum extraction parameters to achieve the highest yield of anthocyanins of 1.326 mg/g from raspberries by using the UADESE were obtained at a water content of 29.24%, ultrasonic power of 209.6 W, extraction temperature of 50.99 °C and extraction time of 31.75 min. Figure 4 describes the best fitness value and level for each factor. The negative sign on the optimal fitness graph is due to the negative sign in the multiple regression equation before running the GA. The best individual plot indicated the extraction time as the best individual. A confirmatory experiment was performed to verify the reliability of the GA. According to the actual situation, the above process parameters were modified as follows: water content of 29%, ultrasonic power of 210 W, extraction temperature of 51 °C and extraction time of 32 min. Under these conditions, the experimental values of the anthocyanin yields were 1.378 ± 0.009 mg/g. The results suggested that the experimental and the predicted values were in close accordance with a 95% confidence interval. Thus, the GA coupled with RSM method was successfully used to optimize the extraction conditions for the UADESE of anthocyanins from raspberries.

### 3.6. Selection of the Two-Phase Solvent System

The partition coefficient (*K*) value in the two-phase solvent system is the critical factor to determine whether the target compounds can be separated. Theoretically, when the *K* value is between 0.5 and 2, the ideal target compounds can be separated from the two-phase solvent system. First, the choice of a two-phase solvent system was measured based on the formation of two layers after mixing: BuOH, MTBE, ACN, water and TFA. In this sense, according to the estimation of the *K* value of the target compound, different ratios of the selected solvents were investigated to optimize the appropriate composition. Table S1 shows that the solvent system No. 2, No. 3 and No. 4 had a suitable range of *K* values 0.5–2.0, which was in-line with the HSCCC solvent system ratios. However, there was no significant difference in the *K* values of different components (I, II, III and IV) (*p >* 0.05), so the four components could not be completely separated. Consequently, the solvent system No. 2, No. 3 and No. 4 were not suitable for use in HSCCC. The solvent system No. 5 had a small *K* value for the compounds (I, II, III and IV), which was not appropriate for separation. The reason for this phenomenon was that the target compound would be washed out quickly when the *K* value was small, which led to the peak’s coincidence of impurity and target. The two-phase solvent system of BuOH-MTBE-ACN-water-TFA (2:2:1:5:0.01, *v*/*v*/*v*/*v*/*v*, NO.1) was an ideal mixture for separation, with reasonable *K* values (from 0.5 to 2) for the four compounds. Hence, NO.1 was selected for HSCCC separation.

### 3.7. Isolation of the Purified Anthocyanin Extract Powders after AB-8 Macroporous Resin Column Chromatography by High-Speed Counter Current Chromatography

**Figure 5:**
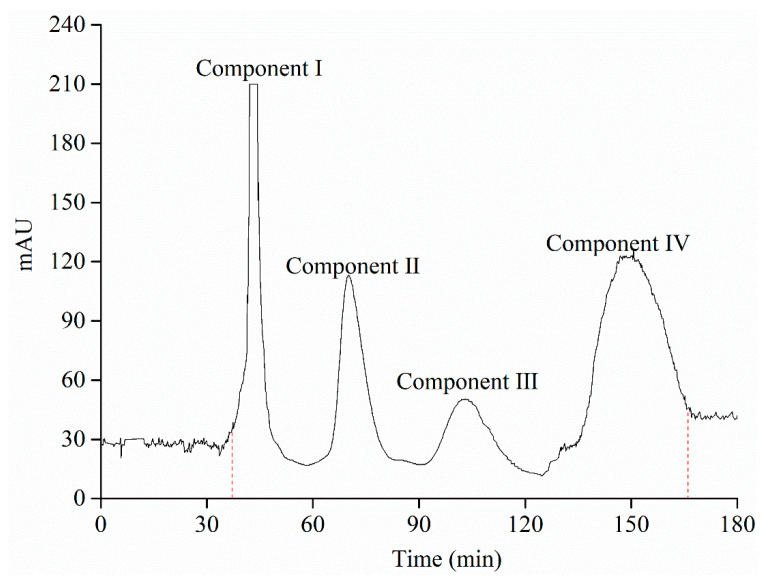
High-speed counter current chromatography (HSCCC) chromatogram of the purified anthocyanin extract samples.

As shown in Figure 5, the four components were isolated by HSCCC. The fractions of component I were collected at the time between 41.3 and 47.5 min, and the fractions of component II were collected at the time between 70.1 min and 73.5 min, whereas fractions concerning component III and component IV were 98.3–109.6 min and 145.5–154.2 min, respectively. Meanwhile, the retention rate of the stationary phase was 48.6%. Component I (25.84 mg; the purity was 78.15% with a recovery of 68.73%), 15.36-mg component II (the purity was 92.25% with a recovery of 83.16%), 12.60-mg component III (the purity was 93.07% with a recovery of 89.61%) and 16.38-mg component IV (the purity was 75.62% with a recovery of 71.39%) were obtained from 300 mg of the purified anthocyanin extract powders after AB-8 macroporous resin column chromatography.

### 3.8. Identification of Separated Peaks

#### 3.8.1. UV-Visible Spectrophotometer Analysis

**Figure 6:**
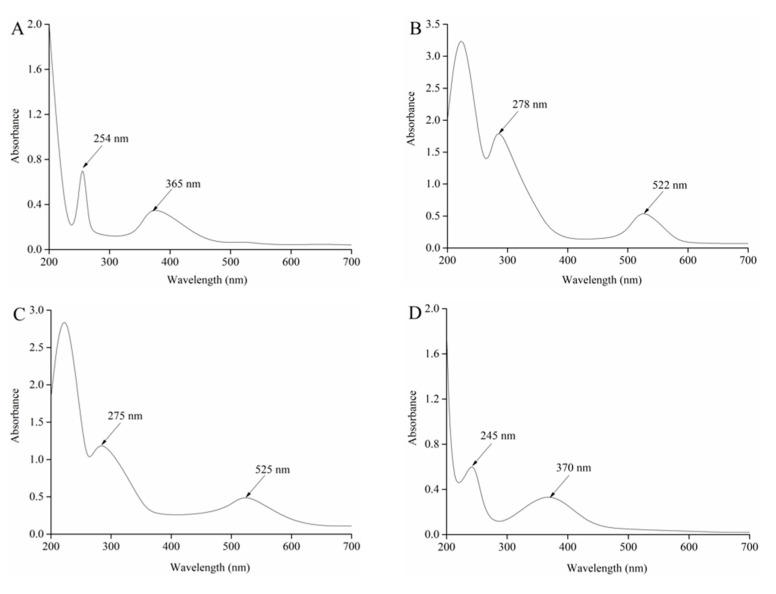
UV-vis spectra of component I (**A**), II (**B**), III (**C**) and IV (**D**) separated from raspberries by HSCCC.

The components I, II, III and IV separated by HSCCC were dissolved in 0.03% KCl solution and diluted to a certain concentration, which were then scanned by UV-Vis spectrometer. The absorption spectra are shown in Figure 6. Anthocyanins belong to polyhydroxy flavonoid compounds and have characteristic absorption peaks at wavelengths of around 280 nm and 520 nm, respectively, while other flavonoids (flavonol, flavonoids, flavanone, etc.) have characteristic absorptions at wavelengths of 240–280 nm and 300–400 nm. Therefore, the absorption characteristics at about 280 nm and 520 nm can be used to determine whether the components are anthocyanins. Figure 6B, C showed that component II and component III had characteristic absorption peaks around 280 nm and 520 nm, indicating that both components were anthocyanins. Component I had characteristic absorption peaks at 254 nm and 365 nm, respectively (Figure 6A), whereas component IV had characteristic absorption peaks at 245 nm and 370 nm, respectively (Figure 6D). The results showed that component I and component IV were non-anthocyanin flavonoid compounds. The main purpose of this study was to isolate and purify anthocyanins from raspberries. Thus, there was no further study on component I and component IV in this paper.

#### 3.8.2. High-Performance Liquid Chromatography-Electrospray Ionization-Mass Spectrometry and Nuclear Magnetic Resonance

**Figure 7:**
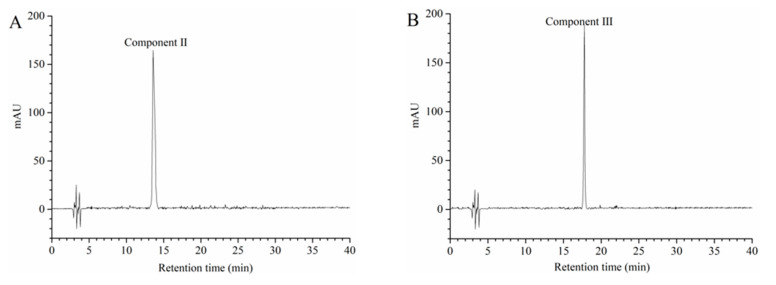
High-performance liquid chromatography (HPLC) chromatogram of component II (**A**) and component III (**B**).

**Figure 8:**
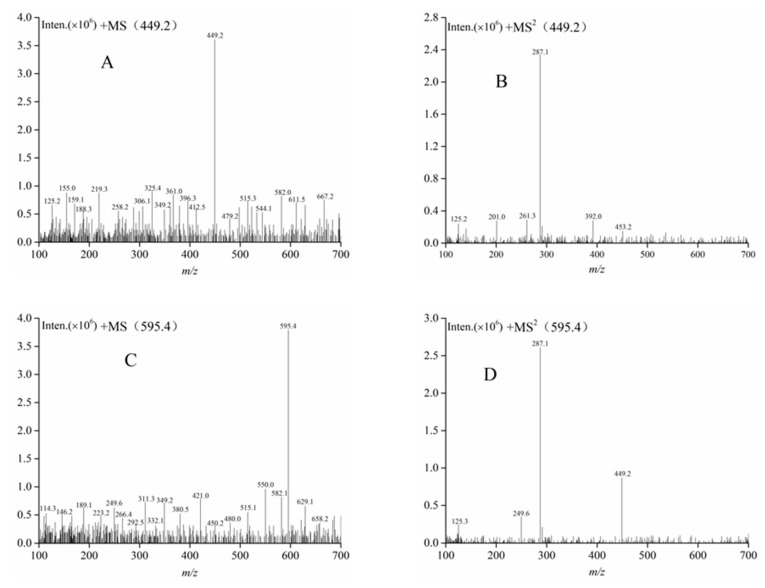
The MS spectra of component II (**A**,**B**) and component III (**C**,**D**).

The chromatographic profiles of the component II and the component III at 520 nm obtained using HPLC-DAD are shown in Figure 7. In the HPLC-DAD analysis, component II and component III had a single peak (Figure 7A,B). The results suggested that component II and component III were an individual anthocyanin, respectively. According to Equation (4), the purities of component II and component III were 92.25% and 93.07%, respectively. Subsequently, HPLC-ESI-MS/MS and NMR were utilized to further identify component II and component III. Identification was carried out by comparing the masses of molecular ions, fragment ions and ^1^H and ^13^C spectra with the results reported in the literature. The data obtained from the analysis of the molecular ion, fragment ion and retention time of anthocyanin peaks by HPLC-ESI-MS/MS are shown in Table 5. The mass spectra of component II and component III are described in Figure 8. The ^1^H and ^13^C spectra of component II and component III are shown in Figures S3–S6, respectively.

The MS analysis of component II (*t*_R_ = 13.58) described the molecular [M]^+^ ion at *m/z* 449.2 (the molecular formula, C_21_H_21_O_11_) and a major fragmentation in MS^2^ at *m/z* 287.1, and the fragment [M-162]^+^ corresponded to the loss of a glucose or galactose moiety (Table 5). The MS^2^ fragmentation of the ion at 287 indicated a cyanidin aglycone moiety. The NMR data is listed as Table S2. These results agreed with those of other studies [48,49]. On the basis of this evidence, component II was finally identified as cyanidin-3-glucoside.

Subsequently, component III (*t*_R_ = 21.69) showed the molecular [M]^+^ ion at *m/z* 595.4 (the molecular formula, C_27_H_31_O_15_). The two MS^2^ fragmentations detected in mass spectrometry were *m/z* 449.2 and *m/z* 287.1, respectively (Table 5). The MS^2^ fragmentation of ion at *m/z* 287.0 corresponded to the cyanidin aglycone moiety. The fragment [M-146-162]^+^ corresponded to the loss of a glycoside on *m/z* 449.0. The NMR data is listed as Table S2. Similarly, the mass spectra and NMR data were obtained by other authors in the case of cyanidin-3-rutinoside from crude mulberry extract and *Liriope plathyphylla*, respectively [50,51]. Hence, component III was finally identified as cyanidin-3-rutinoside.

## 4. Conclusions

This study was performed to investigate the UADESE anthocyanins from raspberries and optimize the extraction conditions by using the GA based on RSM. The optimum extraction parameters to achieve the highest yields of anthocyanins at 1.378 ± 0.009 mg/g from raspberries via UADESE were obtained at a water content of 29%, ultrasonic power of 210 W, extraction temperature of 51 °C and extraction time of 32 min. Under optimal conditions, the purification of anthocyanins by using AB-8 macroporous resin combined with HSCCC techniques produced two main anthocyanins—namely, cyanidin-3-glucoside with a purity of 92.25% and cyanidin-3-rutinoside with a purity of 93.07%. Finally, these findings suggested that the UADESE of the desired anthocyanin components from raspberries was efficient and environmentally friendly.

## Figures and Tables

**Table 1:** Different types of deep eutectic solvents (DESs).

NO.	HBA	HBD	Mole Ratio	DESs:Water (*v*/*v*)
DESs-1	choline chloride	glycerol	1:2	80:20
DESs-2	choline chloride	glycerol	1:3	80:20
DESs-3	choline chloride	1,3-butanediol	1:2	80:20
DESs-4	choline chloride	1,3-butanediol	1:3	80:20
DESs-5	choline chloride	1,4-butanediol	1:2	80:20
DESs-6	choline chloride	1,4-butanediol	1:3	80:20
DESs-7	choline chloride	glycol	1:2	80:20
DESs-8	choline chloride	glycol	1:3	80:20
DESs-9	choline chloride	glucose	1:1	80:20
DESs-10	choline chloride	glucose	1:2	80:20

HBA: hydrogen bond acceptors and HBD: hydrogen bond donors.

**Table 2:** Experimental design independent variables and their levels.

Levels	Independent Variables			
	Water Content (*X*_1_)/%	Ultrasonic Power (*X*_2_)/W	Extraction Temperature (*X*_3_)/°C	Extraction Time (*X*_4_)/min
−1	20	200	45	20
0	30	300	50	30
1	40	400	55	40

**Table 3:** Experimental design and results of response surface methodology (RSM).

No.	Variable				Anthocyanins Yield
	*X*_1_ (%)	*X*_2_ (W)	*X*_3_ (°C)	*X*_4_ (min)	
1	−1	−1	0	0	1.189
2	1	−1	0	0	1.148
3	−1	1	0	0	1.124
4	1	1	0	0	1.121
5	0	0	−1	−1	1.107
6	0	0	1	−1	1.189
7	0	0	−1	1	1.134
8	0	0	1	1	1.204
9	−1	0	0	−1	1.239
10	1	0	0	−1	1.056
11	−1	0	0	1	1.164
12	1	0	0	1	1.205
13	0	−1	−1	0	1.208
14	0	1	−1	0	1.123
15	0	−1	1	0	1.189
16	0	1	1	0	1.254
17	−1	0	−1	0	1.088
18	1	0	−1	0	1.009
19	−1	0	1	0	1.167
20	1	0	1	0	1.098
21	0	−1	0	−1	1.172
22	0	1	0	−1	1.229
23	0	−1	0	1	1.278
24	0	1	0	1	1.248
25	0	0	0	0	1.310
26	0	0	0	0	1.380
27	0	0	0	0	1.299
28	0	0	0	0	1.302
29	0	0	0	0	1.301

**Table 4:** Significance test report of the regression model coefficient of anthocyanins extracted from raspberries by ultrasound-assisted deep eutectic solvent extraction (UADESE).

Source of Variance	*SQ*	*df*	*MS*	*F*-value	*p*-value
Model	0.19	14	0.014	14.84	<0.0001 **
*X*_1_	9.926 × 10^−0.003^	1	9.926 × 10^−0.003^	10.13	0.0066 **
*X*_2_	6.021 × 10^−0.004^	1	6.021 × 10^−0.004^	0.66	0.4314
*X*_3_	0.016	1	0.016	16.95	0.0010 **
*X*_4_	4.840 × 10^−0.003^	1	4.840 × 10^−0.003^	5.28	0.0376 *
*X*_1_^2^	0.097	1	0.097	105.92	<0.0001 **
*X*_2_^2^	9.375 × 10^−0.003^	1	9.375 × 10^−0.003^	10.22	0.0065 **
*X*_3_^2^	0.068	1	0.068	73.77	<0.0001 **
*X*_4_^2^	0.013	1	0.013	14.49	0.0019 **
*X*_1_ *X*_2_	3.610 × 10^−0.004^	1	3.610 × 10^−0.004^	0.39	0.5405
*X*_1_ *X*_3_	2.500 × 10^−0.005^	1	2.500 × 10^−0.005^	0.027	0.8712
*X*_1_ *X*_4_	0.013	1	0.013	13.67	0.0024 **
*X*_2_ *X*_3_	5.625 × 10^−0.003^	1	5.625 × 10^−0.003^	6.13	0.0267 *
*X*_2_ *X*_4_	1.892 × 10^−0.003^	1	1.892 × 10^−0.003^	2.06	0.1729
*X*_3_ *X*_4_	3.600 × 10^−0.005^	1	3.600 × 10^−0.005^	0.039	0.8458
Residual	0.013	14	9.173 × 10^−0.004^		
Lack of fit	7.994 × 10^−0.003^	10	7.994 × 10^−0.004^	0.66	0.7302
Pure error	4.849 × 10^−0.003^	4	1.212 × 10^−0.003^		
Cor total	0.20	28			
	*R*^2^ = 0.9369		*R*^2^_adj_ = 0.8737	*C.V*. = 0.7364	

Note: * stands for significant differences (*p <* 0.05) and ** represents highly significant differences (*p <* 0.01); *SQ* is the sum of squares; *df* is the degrees of freedom and *MS* is the mean square deviation; *C.V*. is the coefficient of variation.

**Table 5:** Identification of anthocyanins in raspberries.

Peaks	Retention Time(min)	Molecular ion (*m/z*)	Molecular Fragments (*m/z*)	Lost Fragments (*m/z*)	Compound
Component II	13.58	449.2	287.1	162	Cyanidin-3-glucoside
Component III	21.69	595.4	449.2, 287.1	146, 162	Cyanidin-3-rutinoside

## Supplementary Materials

The following are available online at https://www.mdpi.com/2304-8158/9/10/1409/s1: Figure S1. The basic process of GA; Figure S2. Diagnostic plots for model adequacy. (A) Predicted versus actual, (B) normal % probability, and (C) internal residuals; Figure S3. ^1^H NMR (400 MHz, CD_3_OD) spectrum of compound II; Figure S4. ^13^C-NMR (100 MHz, CD_3_OD) spectrum of component II; Figure S5. ^1^H NMR (400 MHz, CD_3_OD) spectrum of compound III; Figure S6. ^13^C-NMR (100 MHz, CD_3_OD) spectrum of component III; Table S1. Distribution coefficient (K) of different solvent systems; Table S2. ^1^H, ^13^C-NMR data for component II and component III in CD_3_OD.
