# Supplementary material for: Optimization Ultrasound-Assisted Deep Eutectic Solvent Extraction of Anthocyanins from Raspberry Using Response Surface Methodology Coupled with Genetic Algorithm
Source: Foods. 2020 Oct 4;9(10):1409. doi: 10.3390/foods9101409 (PMC7599779; doi:10.3390/foods9101409)
Supplement: Supplementary file 1 [file foods-09-01409-s001.pdf]

Table of Contents

CONTENTS	page
**Table S1** Distribution coefficient (*K*) of different solvent systems	2
**Table S2** ^{1}H, ^{13}C-NMR data for component II and component III in CD_3OD	3
**Figure S1** The basic process of GA.	4
**Figure S2** Diagnostic plots for model adequacy. (A) predicted versus actual, (B) normal % probability, and (C) internal residuals.	5
**Figure S3** ^{1}H NMR (400 MHz, CD_3OD) spectrum of compound II	6
**Figure S4** ^{13}C-NMR (100 MHz, CD_3OD) spectrum of component II	7
**Figure S5** ^{1}H NMR (400 MHz, CD_3OD) spectrum of compound III	8
**Figure S6** ^{13}C-NMR (100 MHz, CD_3OD) spectrum of component III	9

Table S1. Distribution coefficient (*K*) of different solvent systems.

NO.	BuOH-MTBE-ACN-water-TFA	*K* value			
		Component I	Component II	Component III	Component IV
1	2:2:1:5:0.01	0.62 ± 0.02^{d}	1.89 ± 0.04^{a}	1.15 ± 0.01^{c}	1.61 ± 0.03^{b}
2	3:1:1:5:0.01	0.91 ± 0.02^{a}	0.93 ± 0.01^{a}	0.95 ± 0.02^{a}	0.89 ± 0.01^{a}
3	3:2:1:5:0.01	1.06 ± 0.03^{a}	1.05 ± 0.02^{a}	0.99 ± 0.03^{a}	0.97 ± 0.02^{ab}
4	5:2:1:5:0.01	0.98 ± 0.01^{a}	1.04 ± 0.03^{a}	1.07 ± 0.02^{a}	0.99 ± 0.02^{a}
5	5:3:1:5:0.01	0.42 ± 0.01^{b}	0.83 ± 0.02^{a}	0.40 ± 0.01^{b}	0.98 ± 0.03^{a}

Note: Different lowercase letters (a, b, c, d) in the same line showed significant difference between groups ($p<0.05$).

Table S2. ^{1}H, ^{13}C-NMR data for component II and component III in CD_3OD.

Position	Component II		Component III	
	^{1}H-NMR (400 MHz)	^{13}C-NMR(100 MHz)	^{1}H-NMR (400 MHz)	^{13}C-NMR(100 MHz)
2		162.8		162.8
3		144.2		144.2
4	9.03 (1H, s)	235.3	8.97 (1H, s)	134.7
5		157.8		157.6
6	6.67 (1H, d, *J* =1.8 Hz)	102.2	6.70 (1H, d, *J* =1.8 Hz)	102.0
7		168.9		168.9
8	6.91 (1H, d, *J* =1.8 Hz)	93.6	6.92 (1H, d, *J* =1.8 Hz)	93.7
9		156.2		156.2
10		115.9		115.9
1′		119.8		119.8
2′	8.04 (1H, d, *J* =1.9 Hz)	116.9	8.05 (1H, d, *J* =1.9 Hz)	116.9
3′		146.0		146.0
4′		153.4		154.4
5′	7.02 (1H, dd, *J* =8.8, 1.9 Hz)	111.9	7.05 (1H, dd, *J* =8.7, 1.9 Hz)	111.8
6′	8.28 (1H, d, *J* = 8.8, 1.9 Hz)	126.9	8.31 (1H, d, *J* =8.7, 1.9 Hz)	127.0
1′′	5.33 (1H, d, *J* =7.7 Hz)	101.8	5.31 (1H, d, *J* =7.7 Hz)	101.9
2′′		73.3		73.2
3′′		76.7	3.93~3.37 (8H, m)	76.5
4′′	3.94~3.47 (6H, m)	69.6		69.7
5′′		77.4		75.9
6′′		60.9	4.67 (2H, s,)	66.3
1′′′			4.08 (1H, d, *J* =11.1 Hz)	100.7
2′′′				70.4
3′′′				70.9
4′′′				72.4
5′′′				68.3
6′′′			1.18 (3H, d, *J* =1.5 Hz)	16.4

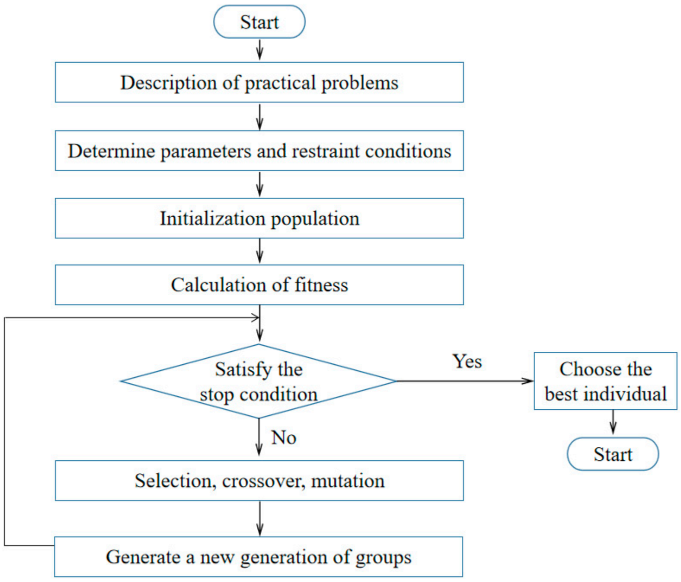

Figure S1 The basic process of GA.

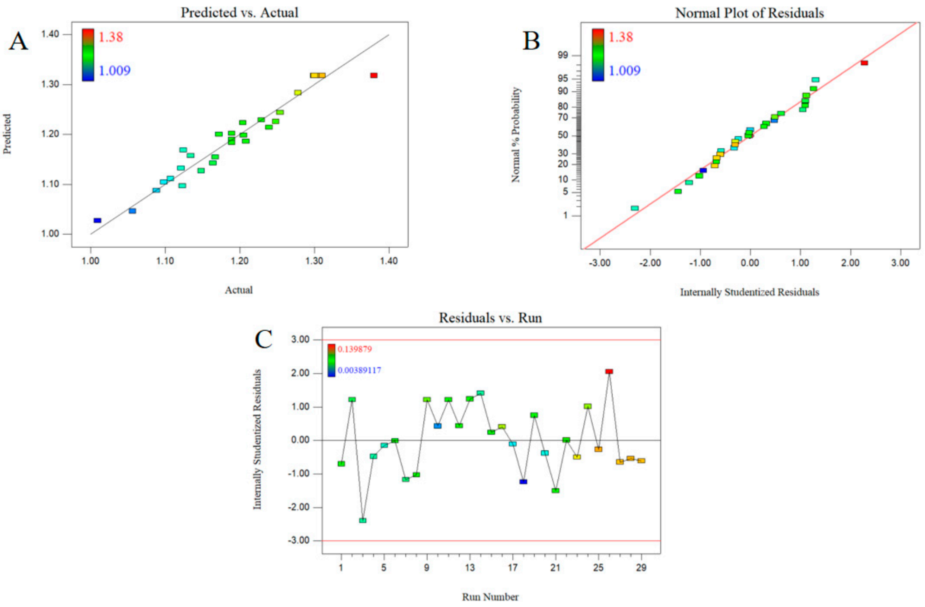

Figure S2 Diagnostic plots for model adequacy. (A) predicted versus actual, (B) normal % probability, and (C) internal residuals.

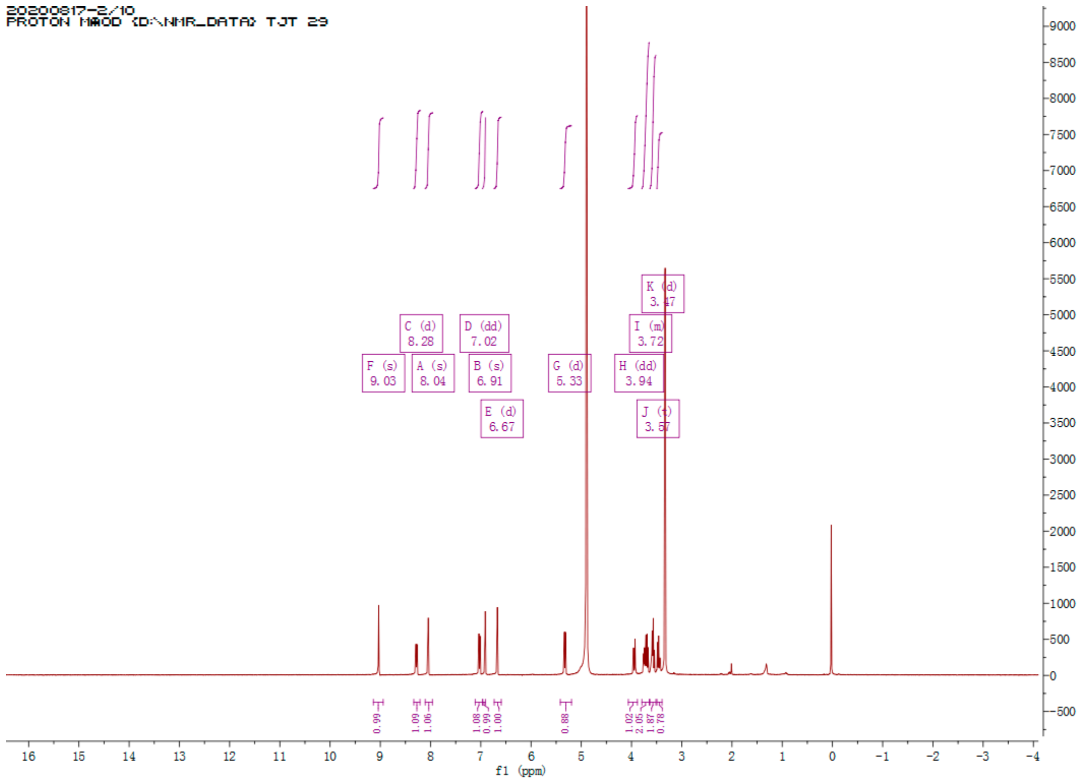

Figure S3 ^{1}H NMR (400 MHz, CD_3OD) spectrum of compound II.

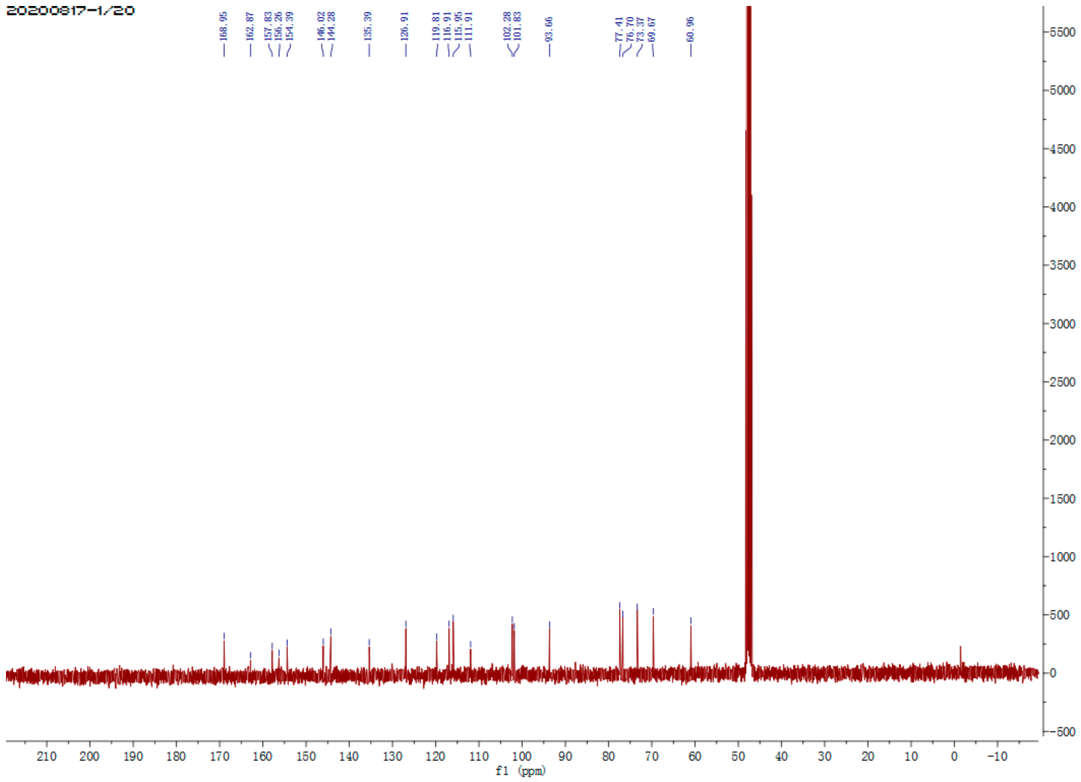

Figure S4 ^{13}C-NMR (100 MHz, CD_3OD) spectrum of component II.

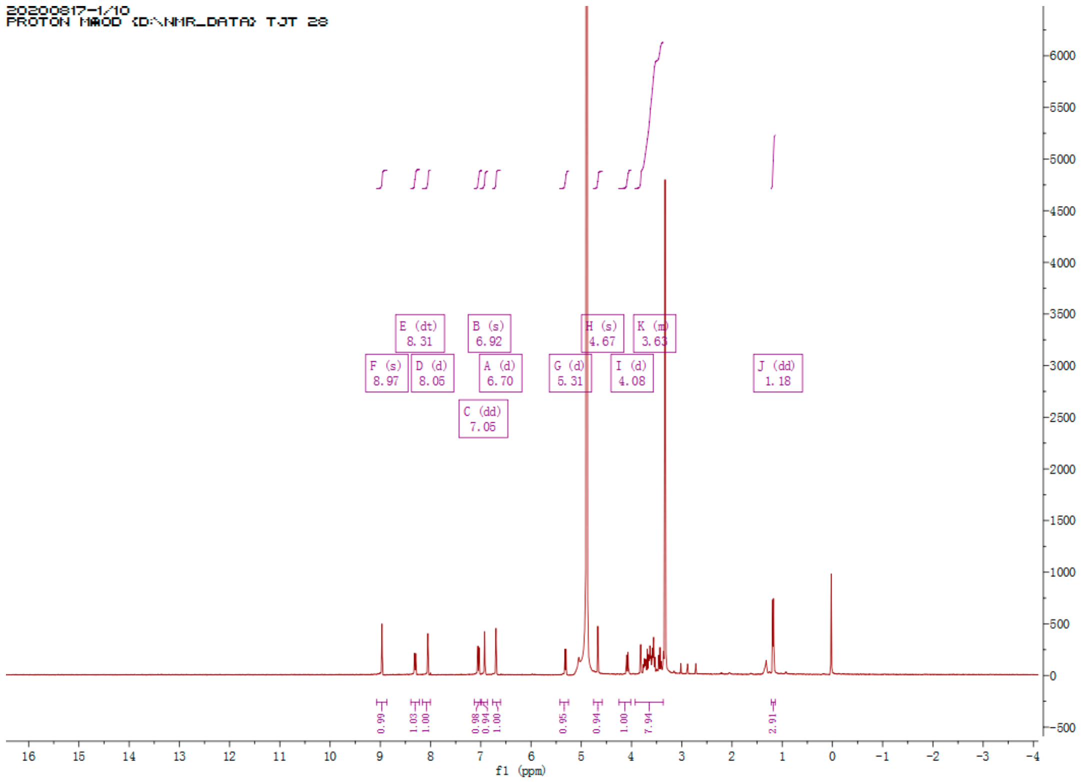

Figure S5 ^{1}H NMR (400 MHz, CD_3OD) spectrum of compound III.

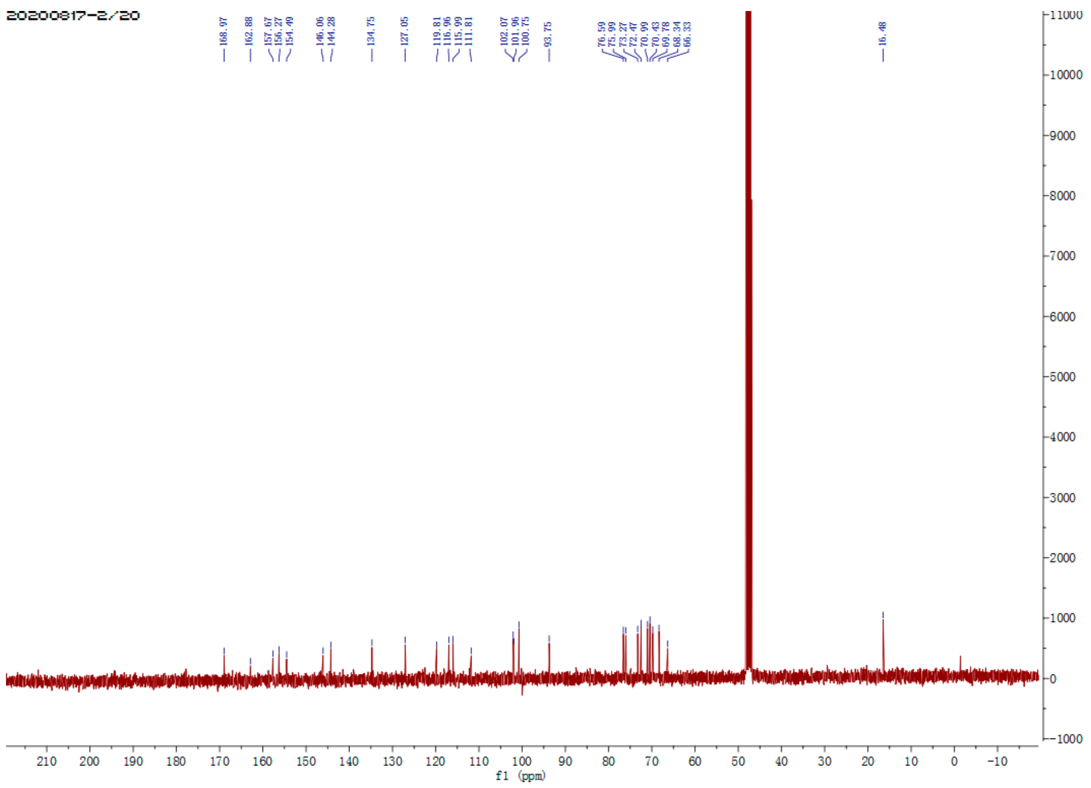

Figure S6 ^{13}C-NMR (100 MHz, CD_3OD) spectrum of component III.
